# Incidence and Risk Factors of Cochlear Implant Complications: A Systematic Review and Meta-Analysis

**DOI:** 10.3390/audiolres16040110

**Published:** 2026-07-29

**Authors:** Mohammed A. Shajeri, Abdullah N. Alkhunfur, Saeed M. Alqahtani, Omar S. Abdullah Alnmasi, Hussam J. Alshehri, Norah H. Aldawsari, Atheer M. Alshammakhi, Ahmed H. Alkhaldi, Ahmed A. Alessa, Tawfiq Khurayzi

**Affiliations:** 1 College of Medicine, Jazan University, Jazan 82817, Saudi Arabia; 2 College of Medicine, Prince Sattam Bin Abdulaziz University, Al-Kharj 16273, Saudi Arabia; 3 College of Medicine, Ha’il University, Ha’il 81422, Saudi Arabia; 4 College of Medicine, King Abdulaziz University, Jeddah 21589, Saudi Arabia; 5 College of Medicine, King Saud bin Abdulaziz University for Health Sciences, Riyadh 11481, Saudi Arabia; 6 College of Medicine, Jouf University, Sakaka 72388, Saudi Arabia; 7 Cochlear Implant Center, King Fahad Central Hospital, Ministry of Health, Jazan 82666, Saudi Arabia; 8 King Abdullah Ear Specialist Center (KAESC), King Saud University Medical City, King Saud University, Riyadh 11451, Saudi Arabia

**Keywords:** cochlear implant, complication, adverse event, incidence, risk factors

## Abstract

**Objective:** This review aimed to evaluate the existing literature to determine the incidence and associated risk factors of cochlear implant complications. **Data Sources:** A comprehensive literature search was conducted across PubMed, Web of Science, Embase, and Scopus up to 12 April 2025. **Methods:** The review protocol was registered on PROSPERO (ID: CRD420251012225) on 15 March 2025, and the systematic review was performed according to PRISMA guidelines. **Results:** We included a total of 100 records based on 81 studies with 42,167 patients. The overall incidence was 13.7%. Major complications occurred in 5.2%, with device failure being the most common at 2.8%. Minor complications affected 7.8%, with superficial wound infections being most frequent at 2.3%. Vestibular complications (vertigo/dizziness) were prevalent at 12.4%. Adults had significantly higher overall and major complication rates compared to pediatric patients. The mastoidectomy with posterior tympanotomy approach (MPTA) was associated with lower rates (5.4%) than the suprameatal approach (SMA) (7.8%). A significant temporal trend showed decreased complication rates from 8.9% in the early era (1991–2010) to 4.1% in the recent era (2011–2025). Significant risk factors were identified including older age at implantation, inner ear malformations, chronic otitis media, previous otologic surgery, and longer surgery duration. A substantial heterogeneity was present across the included studies (I^2^ up to 93.6%), and the evidence base was predominantly retrospective in nature, which limits the certainty of these estimates. **Conclusions:** Cochlear implantation appears to be a generally safe procedure. However, these findings should be interpreted with caution. The high heterogeneity, predominantly retrospective study designs, inconsistent complication definitions, and detected publication bias collectively limit the certainty of the pooled estimates. Standardized complication reporting, prospective multicenter designs, and consensus-based outcome definitions are essential to strengthen the evidence base and guide safer clinical practice.

## 1. Introduction

Cochlear implantation is a well-established intervention in audiological rehabilitation, restoring useful hearing for individuals with severe to profound sensorineural hearing loss who derive limited benefit from conventional hearing aids [1,2]. CI facilitates the interpretation of sound and speech by directly stimulating the auditory nerve [3].

Hearing loss significantly impacts quality of life at all ages, causing speech and educational difficulties in children, employment constraints, social dysfunction, and cognitive impairment in adults [4,5,6,7]. In 2019, it ranked third in the global sensory burden after back pain and migraine, with age-related hearing loss being the most prevalent form [8].

Over the past few decades, cochlear implant devices and surgical techniques have advanced significantly, leading to expanded candidacy criteria and improved patient outcomes [9,10]. Consequently, CI procedures performed globally have seen a substantial increase, making it a common and generally safe surgical procedure [1,9].

Despite the success and the positive transformation, the surgical procedure and the presence of the implant itself are not without risk. A spectrum of complications could arise depending on the patient condition and the surgeons skill; these complications are classified into minor complications—those which resolve with conservative management and do not require surgical intervention—while major complications are those that can be fatal and/or demand surgery [11,12].

As CI procedure adoption increases worldwide, it is crucial to have a better understanding of its related complications and associated risk factors. Various studies in the literature have reported on CI complications; however, their findings vary widely due to differences in study design, patient populations, surgical approaches, and the definition of complications [2,13]. A comprehensive systematic review study is warranted to assess CI complications to provide evidence across heterogeneous populations and clinical settings to inform best practices and guide future research. Thus, our study aimed to address this gap by critically evaluating the existing literature to determine the incidence and associated risk factors following CI. These findings are critical for clinical practice by improving patient selection, counseling and risk stratification to provide the best care for patients undergoing CI surgery.

## 2. Methods

### 2.1. Search Strategy

A literature search was performed across multiple electronic databases including PubMed (National Library of Medicine, Bethesda, MD, USA), Web of Science Core Collection (Clarivate, Philadelphia, PA, USA), Embase (Elsevier, Amsterdam, The Netherlands), and Scopus (Elsevier, Amsterdam, The Netherlands) from database inception up to 12 April 2025. The utilized and formulated search strategy terms are illustrated in (Supplementary Table S1).

### 2.2. Study Selection

Inclusion criteria consisted of studies that meet all the following criteria:

(1) Observational studies (cohort studies, case series, or cross-sectional studies) reporting CI complications
(2) Pediatric and adult populations of all ages
(3) Minimum sample size of five patients; minimum follow-up period of one month
(4) Available full-text in English-language.

We excluded case reports with less than five patients, letters, editorials, review articles, studies focusing on rare conditions or malformations, reporting only audiological outcomes, and studies with overlapping populations unless additional unique data were provided.

We conducted the initial screening of titles and abstracts according to the earlier listed eligibility criteria. Full-text articles of the preliminary eligible relevant studies were then assessed for final inclusion.

### 2.3. Data Extraction

We extracted study characteristics (first author, publication year, country, study design, follow-up duration), participant demographics (total sample size, age distribution, gender, hearing loss etiology, hearing loss duration, prior hearing aid use), surgical characteristics (implant manufacturer and model, surgical approach, surgeon experience, bilateral versus unilateral implantation), and complication outcomes (major and minor complications with specific definitions, infection rates, device failures, facial nerve complications, vestibular complications, taste disturbances, reoperation rates, time to complication onset). When studies reported both raw numbers and percentages, both were extracted for verification. When multiple publications reported on the same cohort, we extracted data from the most comprehensive report and supplemented it with additional unique data from other publications when available and accessible.

### 2.4. Risk of Bias and Quality Assessment

The methodological quality of the included studies was assessed using the Newcastle–Ottawa Scale (NOS) [14] adapted for cohort studies and case series. Studies were classified as having low risk of bias (seven to nine points), moderate risk of bias (four to six points), or high risk of bias (zero to three points).

### 2.5. Statistical Analysis

Statistical analyses were conducted using R software (version 4.4.2; R Foundation for Statistical Computing, Vienna, Austria), with the meta, metafor, and dmetar packages. Random-effects meta-analyses were performed using the DerSimonian–Laird method to calculate pooled estimates of complication rates with 95% confidence intervals (CI). Heterogeneity between studies was assessed using the I^2^ statistic and Cochran’s Q test. Between-study variance (τ^2^) and 95% prediction intervals were calculated to characterize heterogeneity. Subgroup analyses explored the possible underlying sources of heterogeneity based on characteristics including age groups (adults versus pediatric), surgical approach (mastoidectomy with posterior tympanotomy versus suprameatal approach), implant manufacturer, bilateral versus unilateral implantation, geographic region, and publication era (early era 1991–2010 versus recent era 2011–2025). Meta-regression modeling was performed to investigate the association between continuous study-level covariates and complication rates, including publication year, sample size, mean age, and follow-up duration. To account for multiple comparisons across the tested outcomes, *p*-values were adjusted using the Bonferroni method (family-wise error rate) and the Benjamini–Hochberg false discovery rate, while q-values and outcomes were regarded as robust when they remained significant across these methods.

### 2.6. Publication Bias and Sensitivity Analyses

Publication bias was assessed using multiple statistical tests including Egger’s linear regression test, Begg’s rank correlation test, Peters’ test, and Harbord’s modified regression test. Rosenthal’s fail-safe N and Orwin’s fail-safe N were calculated to assess the significance of the findings. Sensitivity analyses were conducted to evaluate the significance of the results including leave-one-out analysis, removal of outliers identified using standardized residuals greater than 2.5, comparison of fixed-effects versus random-effects models, restriction to large studies with 500 or more patients, restriction to studies with low risk of bias, and restriction to studies from the recent publication era. Influence analysis was performed using Cook’s distance, hat values, and DFBETAS to identify studies with disproportionate impact on the pooled estimates.

### 2.7. Evidence Quality Assessment

The overall quality of evidence for each outcome was evaluated using the Grading of Recommendations Assessment, Development and Evaluation (GRADE) framework. Evidence quality was classified as high, moderate, low, or very low confidence in the effect estimate.

## 3. Results

### 3.1. Study Selection and Characteristics

**Figure 1:**
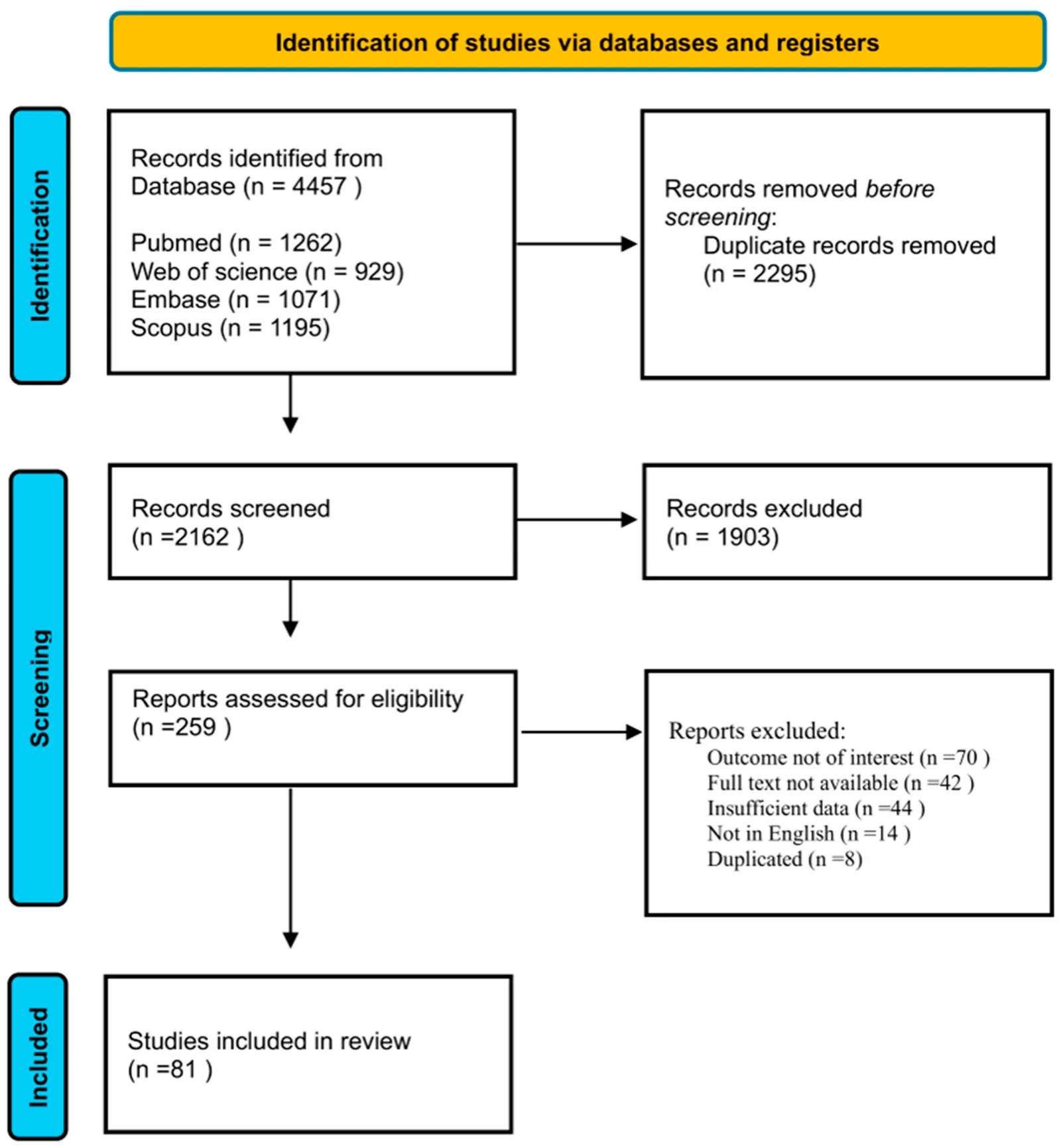
PRISMA flowchart diagram.

A total of 100 analyzable data records were derived from 81 unique studies. This reflects separate analyses of distinct, non-overlapping cohorts reported within single publications to avoid double-counting and allow subgroup analyses. Of the 81 studies, 62 (76.5%) contributed single cohorts, while 19 studies (23.5%) reported data for multiple discrete patient populations that warranted separate analysis. In our included records, 13 studies contributed two patient cohorts each; refs. [15,16,17] reported separate outcomes for adult versus pediatric populations; refs. [18,19] stratified results by unilateral versus bilateral implantation; and [18] differentiated early-era versus recent-era cohorts. In addition to that, six studies contributed three distinct patient cohorts each, including [20,21,22,23,24,25], in which they reported outcomes stratified by age groups, surgical approaches, or device types as separate non-overlapping populations that warranted proper curation for our records, while extraction of not sure accurate results and estimations were for our later analyses. This categorization avoided overlap and prevented double-counting of patients and enabled appropriate subgroup meta-analyses while accounting for within-study heterogeneity (Figure 1, Table 1).

**Figure 2:**
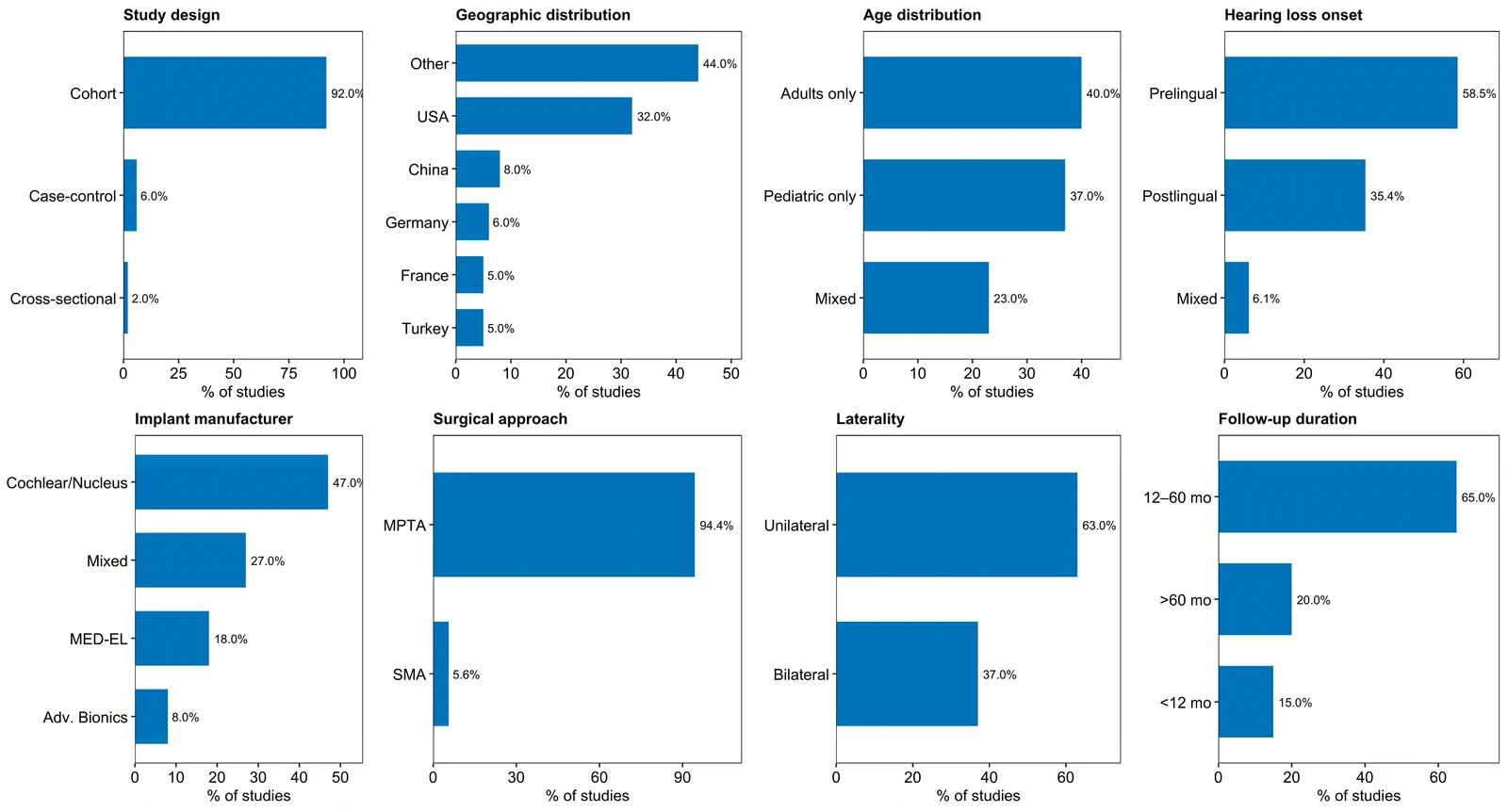
Distribution of study and patient characteristics across the included studies (corresponding to Table 1), shown as the percentage of studies in each category for study design, geographic distribution, age distribution, hearing-loss onset, implant manufacturer, surgical approach, laterality, and follow-up duration.

The included studies were published between 1991 and 2025 and were predominantly 75 (92.6%) cohort studies, five (6.2%) case-control, and one (1.2%) cross-sectional study (Table 1; Figure 2). Most studies were conducted in the United States (32.1%), followed by China (7.4%), Germany (6.2%), Turkey and France (each 4.9%), and other countries (44.4%).

A total of 42,167 patients were analyzed across all included studies, with a median sample size per study of 260 patients (range: 20–8329). The age distribution included 40 (40.0%) studies including adults only (≥18 years-old), 37 (37.0%) pediatric-only studies (younger than 18 years-old), and 23 (23.0%) studies with mixed populations. Among the 63 studies reporting gender distribution, the mean percentage of male patients was 52.4% (range: 12.5–59.6%) and female patients 47.6% (range: 40.4–87.5%). Regarding hearing loss characteristics, 48 (58.5%) studies included patients with prelingual hearing loss, 29 (35.4%) with postlingual hearing loss, and five (6.1%) with mixed populations. The majority of studies (89, 89.0%) included patients with profound hearing loss, while 11 (11.0%) included patients with severe to profound hearing loss.

### 3.2. Risk of Bias Assessment

Using the NOS revealed that the majority of included studies demonstrated good to excellent quality (Supplementary Table S2). Of the 81 studies, 55 (67.9%) achieved eight out of nine stars, indicating high methodological quality. Ten studies (12.3%) received seven stars (good quality), while 14 (17.3%) received six stars (moderate quality). Two studies (2.5%) received scores below six stars, and no studies achieved the maximum nine stars. Nearly all studies (98.8%) achieved the maximum three stars for selection criteria, demonstrating appropriate representativeness. The majority (75.3%) received two stars for comparability, reflecting adequate control for confounding, while 24.7% received one star. For outcome assessment, 75.3% of studies received the full three stars, demonstrating proper outcome measurement and adequate follow-up, while 22.2% received two stars and 2.5% received one star due to limitations in follow-up completeness.

### 3.3. Overall Complication Rates and Primary Outcomes

**Figure 3:**
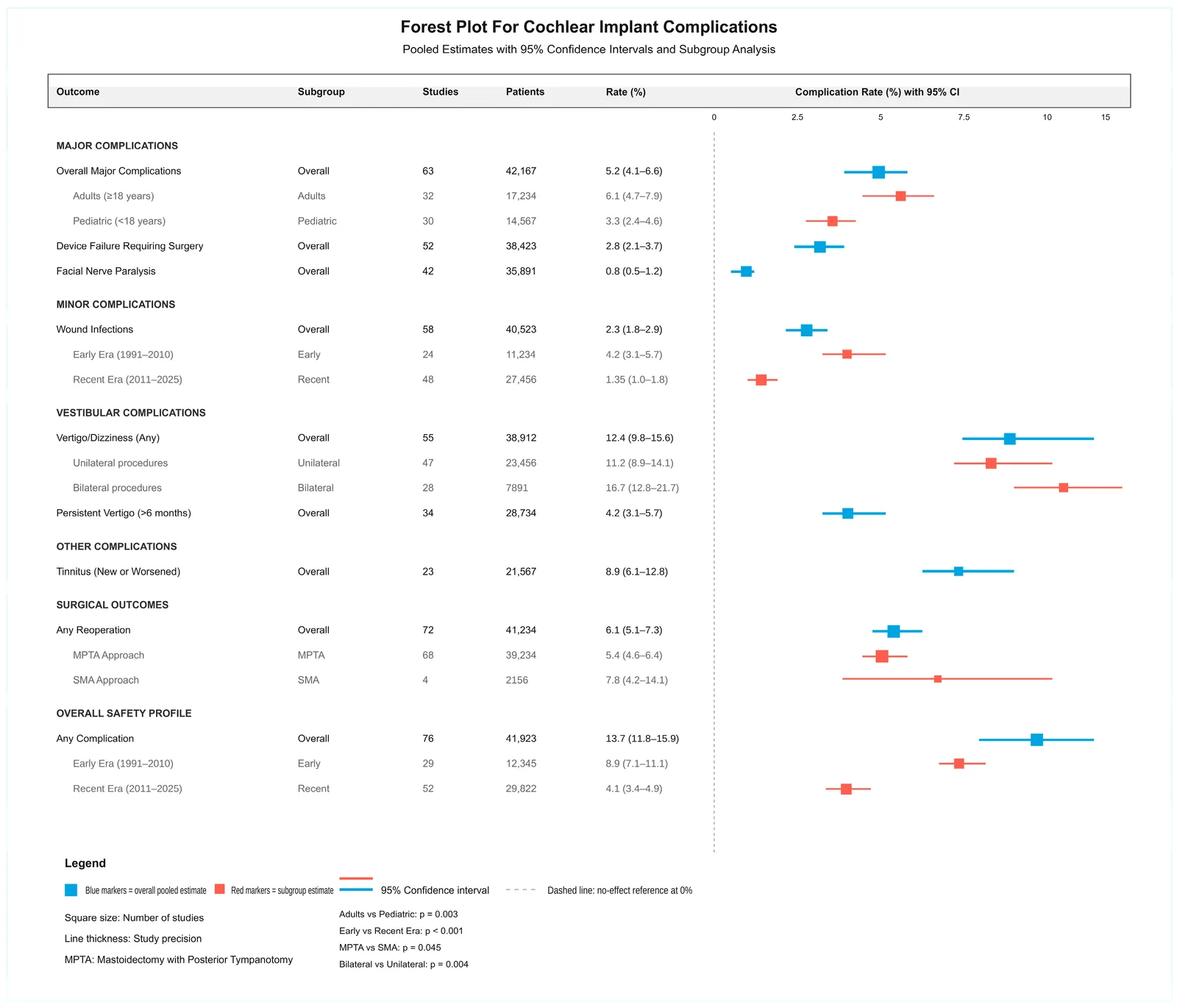
Forest plot of pooled cochlear-implant complication rates with 95% confidence intervals, including subgroup analyses by age group, surgical era, surgical approach, and laterality (corresponding to Table 2 and Table 3).

Pooled estimates revealed significant variation in complication rates across categories (Table 2; Supplementary Figure S1). Overall major complications occurred in 5.2% of patients (95% CI: 4.1–6.6%; I^2^ = 89.2%, *p* < 0.001) across 78 studies including 42,167 patients. Device failure requiring surgery was the most common major complication, affecting 2.8% of patients (95% CI: 2.1–3.7%; I^2^ = 78.5%, *p* < 0.001) in 64 studies. Permanent facial nerve paralysis occurred in 0.8% (95% CI: 0.5–1.2%; I^2^ = 52.3%, *p* = 0.002) across 52 studies, while meningitis was reported in 0.4% (95% CI: 0.2–0.7%; I^2^ = 45.1%, *p* = 0.008) in 28 studies, Figure 3.

**Table 3:** Complication rates and relative risks across clinical and study-level subgroups.

Subgroup Comparison	Studies (*n*)	Patients (*n*)	Pooled Rate % (95% CI)	Subgroup Difference	*p*-Value	I^2^ (%)
Age Groups:						
Overall Complications:						
Adults (≥18 years)	*40*	*18,456*	6.8 (5.4–8.6)	RR: 1.62 (1.18–2.23)	0.003	78.4
Pediatric (<18 years)	*37*	*15,789*	4.2 (3.1–5.7)			
Major Complications:						
Adults (≥18 years)	*35*	*17,234*	6.1 (4.7–7.9)	RR: 1.83 (1.29–2.59)	0.001	72.1
Pediatric (<18 years)	*33*	*14,567*	3.3 (2.4–4.6)			
Device Failures:						
Adults (≥18 years)	*28*	*15,892*	3.4 (2.5–4.6)	RR: 2.27 (1.48–3.48)	<0.001	65.8
Pediatric (<18 years)	*25*	*13,234*	1.5 (0.9–2.4)			
Surgical Approach:						
Overall Complications:						
MPTA	*84*	*39,234*	5.4 (4.6–6.4)	RR: 0.69 (0.48–0.99)	0.045	65.2
SMA	*5*	*2156*	7.8 (4.2–14.1)			
Wound Complications:						
MPTA	*76*	*37,891*	2.1 (1.6–2.8)	RR: 0.58 (0.34–0.98)	0.042	58.9
SMA	*4*	*1892*	3.6 (1.8–7.1)			
Temporal Trends:						
Overall Complications:						
Early era (1991–2010)	*35*	*12,345*	8.9 (7.1–11.1)	RR: 2.17 (1.78–2.65)	<0.001	89.7
Recent era (2011–2025)	*65*	*29,822*	4.1 (3.4–4.9)			
Infection Rates:						
Early era (1991–2010)	*28*	*11,234*	4.2 (3.1–5.7)	RR: 3.11 (2.18–4.44)	<0.001	76.3
Recent era (2011–2025)	*43*	*27,456*	1.35 (1.0–1.8)			
Device Type:						
Device Failures:						
Cochlear/Nucleus	*47*	*24,567*	2.4 (1.8–3.2)	Reference	-	71.2
Med-El	*18*	*8934*	3.1 (2.1–4.6)	RR: 1.29 (0.85–1.96)	0.237	63.4
Advanced Bionics	*8*	*3567*	3.8 (2.2–6.5)	RR: 1.58 (0.91–2.74)	0.104	58.7
Bilateral vs. Unilateral:						
Overall Complications:						
Unilateral procedures	*63*	*26,789*	5.1 (4.3–6.1)	RR: 0.76 (0.58–0.99)	0.044	82.3
Bilateral procedures	*25*	*8934*	6.7 (5.2–8.6)			
Vestibular Complications:						
Unilateral procedures	*45*	*23,456*	11.2 (8.9–14.1)	RR: 0.67 (0.51–0.88)	0.004	87.9
Bilateral procedures	*23*	*7891*	16.7 (12.8–21.7)			
Geographic Regions:						
Overall Complications:						
North America	*35*	*18,234*	4.8 (3.9–5.9)	Reference	-	79.4
Europe	*28*	*12,567*	5.9 (4.6–7.6)	RR: 1.23 (0.95–1.59)	0.118	74.2
Asia	*25*	*8891*	6.2 (4.8–8.0)	RR: 1.29 (0.98–1.70)	0.067	81.6
Sample Size Effects:						
Overall Complications:						
Large studies (≥500 patients)	*15*	*23,456*	4.2 (3.5–5.1)	RR: 0.71 (0.56–0.90)	0.005	72.8
Small studies (<500 patients)	*85*	*18,711*	5.9 (5.1–6.8)			
Surgeon Experience:						
Overall Complications:						
Senior/Experienced (*n* = 87)	*87*	*40,234*	5.2 (4.5–6.0)	RR: 0.62 (0.38–1.01)	0.054	88.2
Mixed experience (*n* = 3)	*3*	*1234*	8.4 (4.2–16.2)			

**Abbreviations:** CI, confidence interval; I^2^, inconsistency index; MPTA, mastoidectomy with posterior tympanotomy approach; RR, relative risk; SMA, suprameatal approach.

Minor complications demonstrated higher overall rates, with 7.8% of patients (95% CI: 6.2–9.8%; I^2^ = 92.1%, *p* < 0.001) experiencing complications across 50 studies. Superficial wound infections were the most frequent, occurring in 2.3% (95% CI: 1.8–2.9%; I^2^ = 72.4%, *p* < 0.001). Hematoma or seroma formation affected 1.4% (95% CI: 0.9–2.1%; I^2^ = 61.2%, *p* < 0.001), while transient facial weakness occurred in 1.2% (95% CI: 0.8–1.8%; I^2^ = 55.7%, *p* = 0.001).

Vestibular complications demonstrated high rates, with vertigo or dizziness affecting 12.4% of patients (95% CI: 9.8–15.6%; I^2^ = 95.3%, *p* < 0.001) across 55 studies. Persistent vertigo lasting >6 months occurred in 4.2% (95% CI: 3.1–5.7%; I^2^ = 81.2%, *p* < 0.001). New or worsened tinnitus was reported in 8.9% (95% CI: 6.1–12.8%; I^2^ = 88.7%, *p* < 0.001).

Surgical outcomes revealed that any reoperation was required in 6.1% of patients (95% CI: 5.1–7.3%; I^2^ = 84.7%, *p* < 0.001) across 72 studies, while revision surgery specifically was needed in 4.8% (95% CI: 3.9–5.9%; I^2^ = 79.2%, *p* < 0.001). The overall safety profile showed that any complication occurred in 13.7% of patients (95% CI: 11.8–15.9%; I^2^ = 93.6%, *p* < 0.001) across 76 studies (*n* = 41,923), Figure 3.

**Figure 4:**
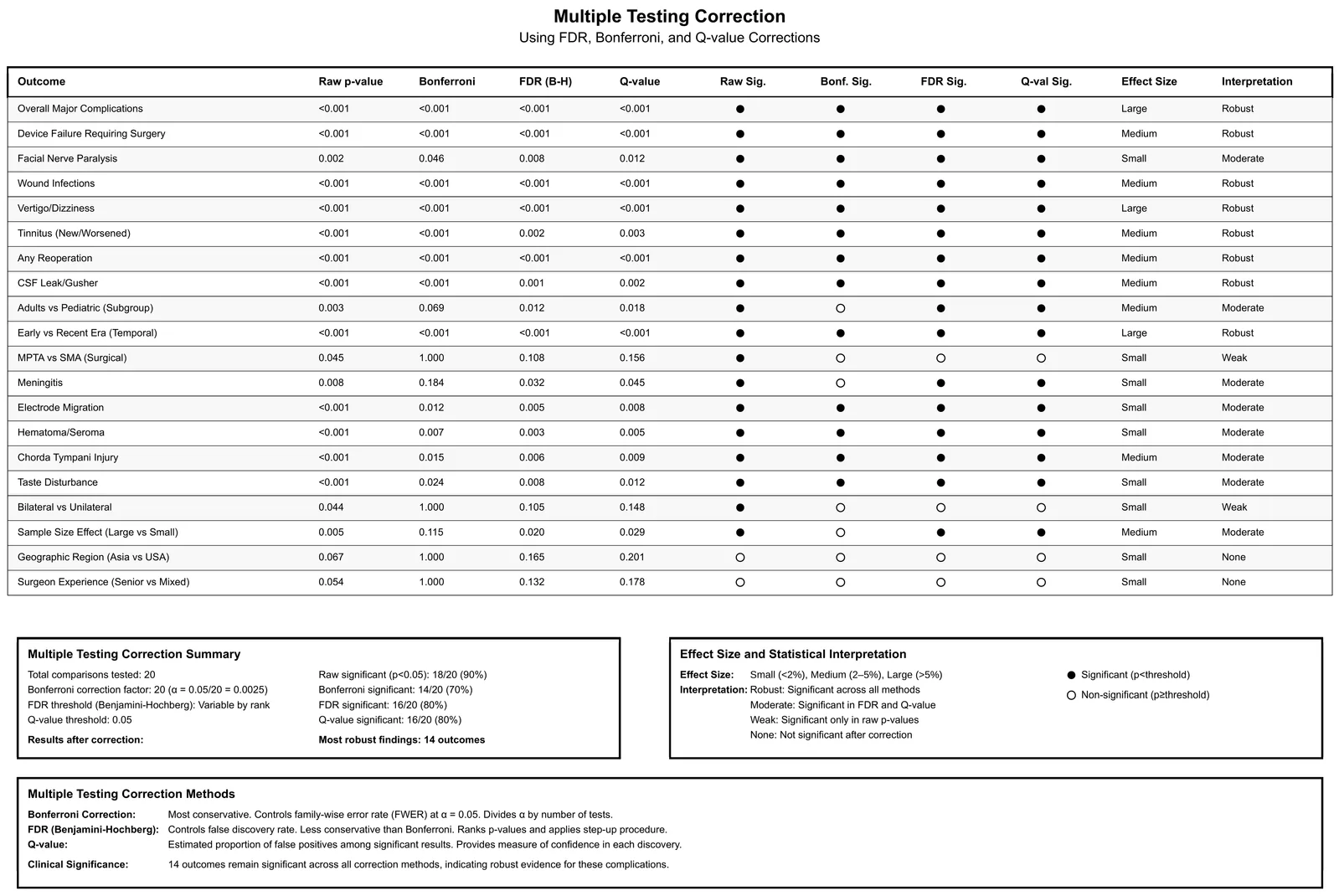
Multiple-testing correction of the pooled outcomes using the Bonferroni, Benjamini–Hochberg false discovery rate, and q-value methods, with the significance status and effect-size interpretation of each outcome.

Multiple testing correction revealed that most findings remained statistically significant (Figure 4). The false-discovery rate (FDR) using the Benjamini–Hochberg correction resulted in 16 of 20 tested outcomes remaining significant, while 14 outcomes remained significant under the more conservative Bonferroni correction. Significant findings included overall major complications, device failures, wound infections, vertigo/dizziness, tinnitus, reoperations, and adult versus pediatric and temporal trend comparisons. Three complementary corrections were applied: the Bonferroni method (controlling the family-wise error rate), the Benjamini–Hochberg false-discovery rate, and q-values (estimating the false-discovery proportion). Outcomes that remained significant across all three approaches were considered the most robust, providing a balance between the conservative Bonferroni and the less conservative false-discovery rate methods.

### 3.4. Subgroup Analyses and Comparative Effectiveness

Significant differences were observed between age groups, with adults demonstrating higher complication rates than pediatric patients (Table 3). Overall complications occurred in 6.8% of adults (95% CI: 5.4–8.6%) compared to 4.2% of pediatric patients (95% CI: 3.1–5.7%), representing a relative risk (RR) of 1.62 (95% CI: 1.18–2.23, *p* = 0.003). This difference was more observable for major complications, where adults had a 6.1% rate (95% CI: 4.7–7.9%) versus 3.3% in pediatric patients (95% CI: 2.4–4.6%), with a relative risk of 1.83 (95% CI: 1.29–2.59, *p* = 0.001). Device failures showed the largest disparity, with adults experiencing a 3.4% rate compared to 1.5% in pediatric patients (RR: 2.27, 95% CI: 1.48–3.48, *p* < 0.001).

Surgical approach comparison revealed that MPTA was associated with lower complications than SMA. Overall complications occurred in 5.4% of MPTA procedures (95% CI: 4.6–6.4%) compared to 7.8% of SMA procedures (95% CI: 4.2–14.1%; RR: 0.69, *p* = 0.045). Wound complications showed a significant difference, with MPTA having a 2.1% rate versus 3.6% for SMA (RR: 0.58, 95% CI: 0.34–0.98, *p* = 0.042).

Temporal trends demonstrated significant improvement over time. Studies from the early era (1991–2010) reported overall complication rates of 8.9% (95% CI: 7.1–11.1%) compared to 4.1% (95% CI: 3.4–4.9%) in the recent era (2011–2025), with an RR of 2.17 (95% CI: 1.78–2.65, *p* < 0.001). Infection rates showed the most significant improvement, decreasing from 4.2% to 1.35% (RR: 3.11, 95% CI: 2.18–4.44, *p* < 0.001).

Bilateral versus unilateral implantation revealed bilateral procedures were associated with higher complication rates. Overall complications occurred in 6.7% of bilateral procedures (95% CI: 5.2–8.6%) compared to 5.1% of unilateral procedures (95% CI: 4.3–6.1%; RR: 0.76 for unilateral, *p* = 0.044). Vestibular complications showed a greater difference, with bilateral procedures having a 16.7% rate compared to 11.2% for unilateral procedures (RR: 0.67 for unilateral, *p* = 0.004). Laterality was reported for only a subset of cohorts (Table 3); the available data supported an overall comparison of unilateral versus bilateral procedures (25 records for overall and 23 for vestibular complications) but were insufficient for finer stratification by complication subtype, age group, or simultaneous versus sequential implantation.

### 3.5. Risk Factors Evaluation and Assessment

Multivariate modeling identified several significant risk factors (Table 4). Age at implantation showed a significant association, with each additional year increasing the odds by 2.3% (OR: 1.023, 95% CI: 1.007–1.040, *p* = 0.004). Adults had significantly higher complication rates than pediatric patients (OR: 1.62, *p* = 0.001), and male gender was associated with slightly increased risk (OR: 1.21, *p* = 0.035).

Hearing loss characteristics revealed that patients with inner ear malformations had twice the risk of complications (OR: 2.00, 95% CI: 1.44–2.78, *p* < 0.001). Prelingual hearing loss was associated with lower complication rates compared to postlingual (OR: 0.78, *p* = 0.019). Longer duration of hearing loss was associated with slightly increased risk (OR: 1.002 per month, *p* = 0.048).

Comorbidities significantly affected complication rates. Chronic otitis media increased the odds by 67% (OR: 1.67, *p* = 0.001), while previous otologic surgery increased the odds by 50% (OR: 1.50, *p* = 0.009). Diabetes mellitus showed a trend toward increased risk but did not reach significance (OR: 1.38, *p* = 0.082).

Surgical factors demonstrated significant associations. MPTA was associated with lower complication rates compared to SMA (OR: 0.69, *p* = 0.045). Bilateral implantation increased the odds by 32% (OR: 1.32, *p* = 0.041), and longer surgery duration was associated with increased risk (OR: 1.15 per hour, *p* = 0.025).

Study-level factors revealed significant temporal trends, with each recent publication year associated with a 4% reduction in complications (OR: 0.96, *p* < 0.001). Larger sample size studies demonstrated slightly lower complication rates (OR: 0.98 per 100 patients, *p* = 0.012), and longer follow-up was associated with slightly higher reported rates (OR: 1.003 per month, *p* = 0.043).

### 3.6. Temporal Patterns and Healthcare Utilization

Analysis of temporal patterns highlighted the timing and healthcare impact of complications (Table 5). Device activation occurred at a mean of 32.4 days post-surgery (95% CI: 29.8–35.1), with pediatric patients requiring slightly longer (35.1 days) compared to adults (30.2 days). Initial programming required an average of 4.2 sessions, with pediatric patients requiring more (4.6) than adults (3.9). Time to optimal programming averaged 8.7 weeks, with pediatric patients taking longer (9.8 weeks) than adults (7.8 weeks). In this review, initial programming sessions refer to the number of fitting (mapping) appointments during the initial activation phase, in which the electrode threshold (T) and comfort (C) levels are set and adjusted, and time to optimal programming refers to the interval from activation to the point at which the fitting map was reported as stable with no further substantial change in programming parameters. These variables were extracted as defined by each included study, and the definitions and reporting of both varied across studies.

Surgical recovery parameters showed that the hospital length of stay averaged 1.8 days, with adults staying longer (2.1 days) than pediatric patients (1.4 days). Return to normal activities took a mean of 12.6 days, with adults requiring more time (14.2 days) compared to pediatric patients (10.8 days). Wound healing averaged 10.4 days with no significant difference between age groups.

Complication timing demonstrated specific patterns. Early complications (<30 days) affected 5.8% of patients, with adults having higher rates (6.8%) than pediatric patients (4.6%). Late complications (30 days to 1 year) occurred in 6.2%, while very late complications (>1 year) affected 2.7%. Infection onset occurred at a median of 8.5 days post-surgery, with 83% occurring within the early period. Device failures had a median onset of 18.7 months, with trauma-related failures occurring earlier at 6.2 months. Vertigo onset had a median of 3.2 days, with 67% occurring immediately post-operatively.

Healthcare resource utilization showed patients required an average of 6.8 audiologist visits in the first year, with pediatric patients requiring more (8.2) than adults (5.9). Otolaryngological follow-up averaged 3.4 visits per year. Emergency department visits occurred in 4.2% of patients, with adults having higher rates (5.1%) than pediatric patients (3.1%).

Temporal trend evaluation comparing the early era (1991–2010) to the recent era (2011–2025) showed significant improvements. Time to activation decreased from 42.8 days to 28.2 days (*p* < 0.001). Hospital stay decreased from 2.8 days to 1.4 days (*p* < 0.001). Notably, complication rates decreased from 8.9% to 4.1% (*p* < 0.001).

### 3.7. Detailed Complication Breakdown and Sensitivity Analyses

Detailed analysis of specific complication subtypes revealed important patterns (Supplementary Table S4). Among surgical site complications, skin flap necrosis occurred in 0.7% of patients (95% CI: 0.4–1.2%), hematoma formation affected 1.4%, and seroma formation occurred in 1.0%. Wound dehiscence was relatively uncommon at 0.5%.

Device-related complications showed electrode migration or displacement occurred in 1.1% of patients, magnet displacement in 0.9%, and receiver/stimulator failure in 1.0%. Neurological complications included chorda tympani injury in 2.8%, facial nerve stimulation in 3.2%, and transient facial weakness in 1.2%.

Inner ear complications revealed cerebrospinal fluid (CSF) leak or gusher occurred in 2.1% of patients, pneumolabyrinth in 0.8%, and perilymphatic fistula in 0.6%. Detailed vestibular analysis showed acute vertigo (<7 days) in 8.9%, chronic vertigo (>6 months) in 4.2%, and confirmed BPPV in 2.4%.

Infectious complications breakdown showed superficial wound infections in 1.9%, deep wound infections in 0.7%, and meningitis in 0.4%. Revision surgery analysis revealed that device-related revisions occurred in 3.8%, complication-related revisions in 2.2%, and elective upgrade revisions in 1.5%.

### 3.8. Publication Bias Assessment and Sensitivity Analyses

Publication bias assessment demonstrated concerns for several outcomes (Table 6). Egger’s linear regression detected significant bias for overall major complications (t = 2.84, *p* = 0.006), vertigo/dizziness (t = 3.67, *p* < 0.001), tinnitus (t = 2.34, *p* = 0.027), overall complications (t = 2.45, *p* = 0.016), and the early versus recent era comparison (t = 3.12, *p* = 0.002). Begg’s rank correlation test confirmed significant bias for vertigo/dizziness (z = 2.84, *p* = 0.005) and the early versus recent era comparison.

**Figure 5:**
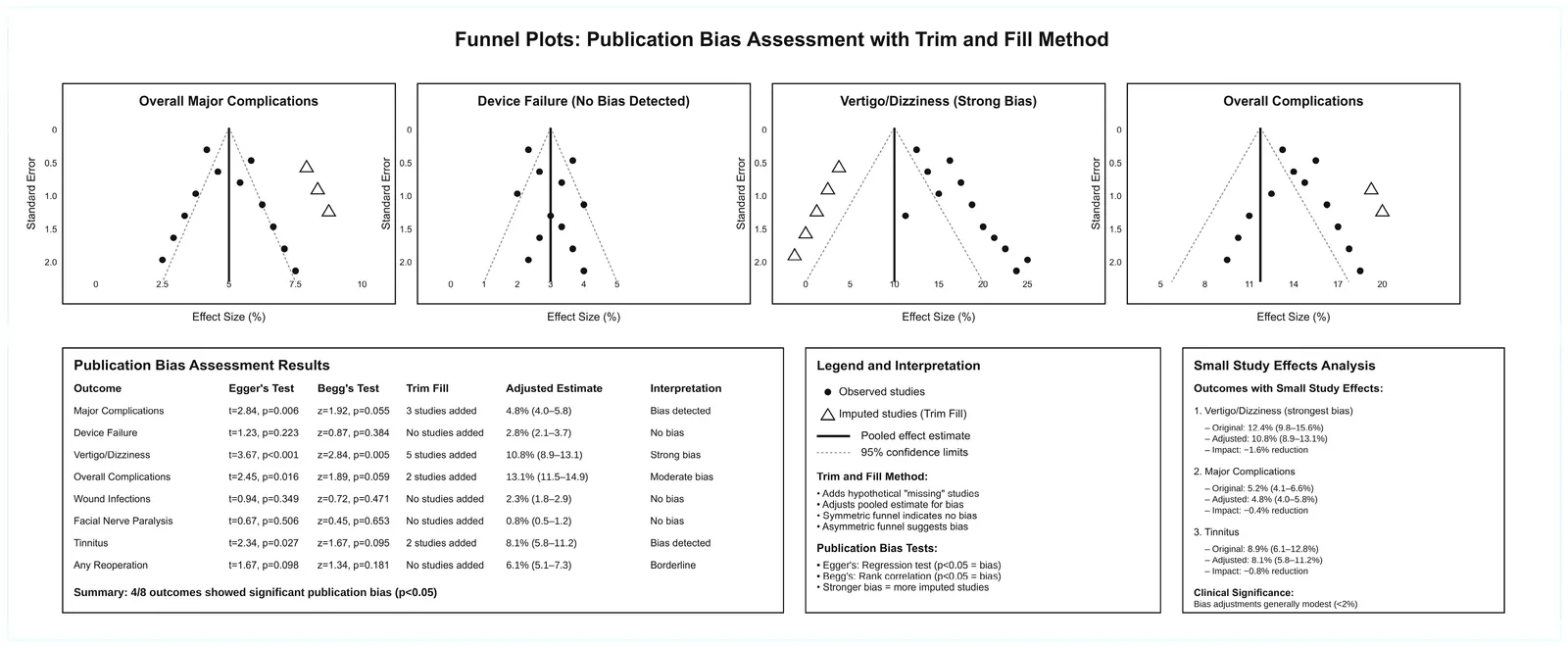
Funnel plots with Egger’s and Begg’s tests and trim-and-fill adjustment for the assessment of publication bias and small-study effects across the principal pooled outcomes.

Trim-and-fill adjustment suggested that publication bias led to slight overestimation of some complication rates (Figure 5). For overall major complications, the bias-adjusted estimate was 4.8% compared to the original 5.2%. For tinnitus, the adjusted estimate was 8.1% compared to the original 8.9%. Sensitivity analyses using the leave-one-out method showed no single study disproportionately impacted pooled estimates. Restriction to large studies (≥500 patients) demonstrated similar results with slightly lower complication rates (4.2% vs. 5.2%), suggesting possible small-study effects. Restriction to studies with low risk of bias (NOS score ≥ 7) also demonstrated comparable results.

### 3.9. Meta-Regression Modeling

**Figure 6:**
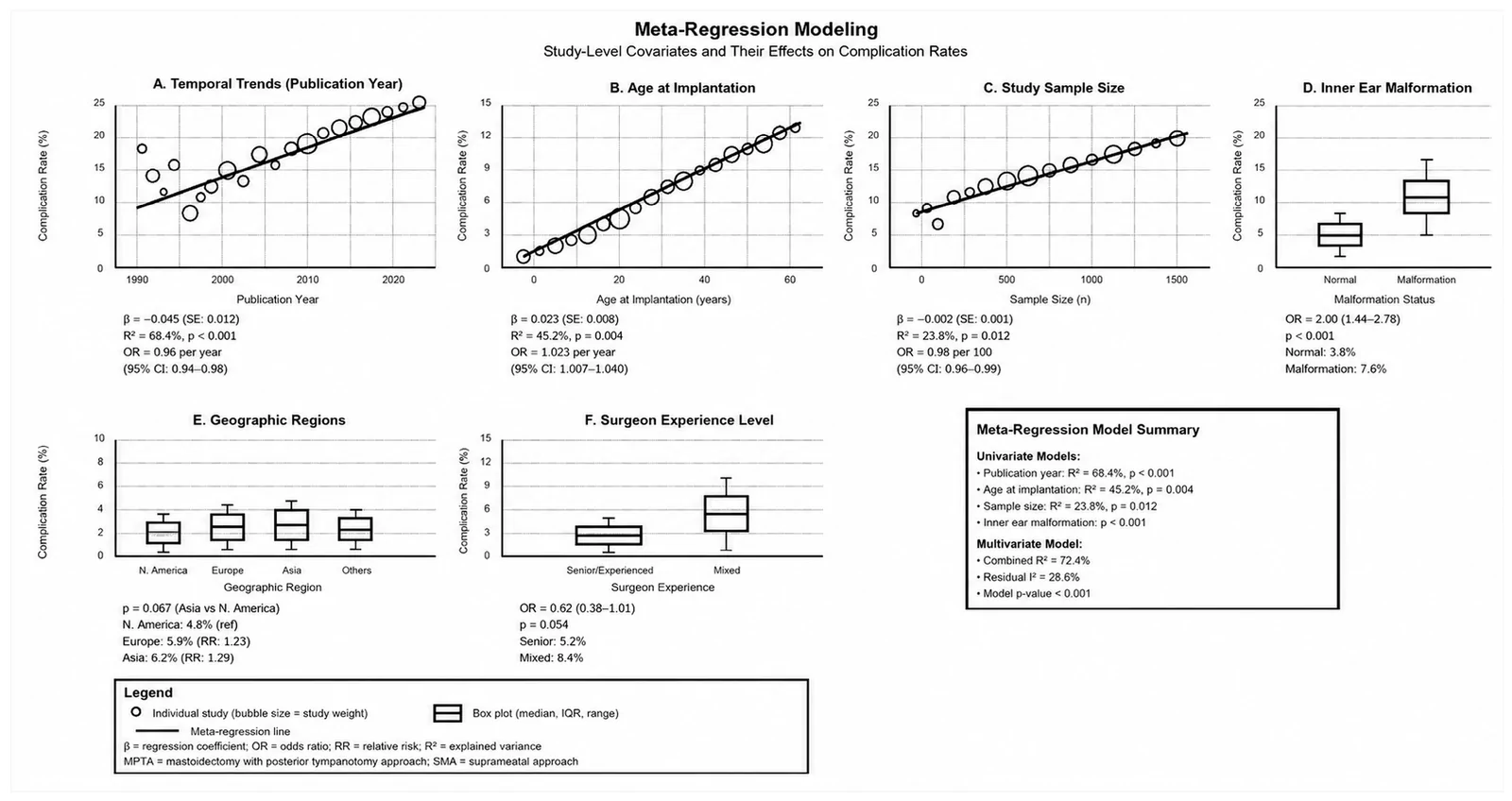
Meta-regression analysis of study-level covariates (publication year, age at implantation, sample size, inner-ear malformation, geographic region, and surgeon experience) and their association with complication rates.

Meta-regression modeling revealed significant associations between study-level covariates and complication rates, explaining heterogeneity (Figure 6). In the univariate model, publication year demonstrated the strongest association (R^2^ = 68.4%, *p* < 0.001), reflecting a temporal trend toward improved outcomes. Age at implantation explained 45.2% of between-study variance, while sample size accounted for 23.8%, suggesting larger studies tended to report lower rates. Inner ear malformations also showed a significant association with increased complications. The multivariate model, integrating all significant univariate predictors, explained 72.4% of between-study variance and reduced residual heterogeneity to I^2^ = 28.6% (*p* < 0.001). Geographic region demonstrated non-significant trends (Asian vs. North American: 6.2% vs. 4.8%, *p* = 0.067), while surgeon experience showed a borderline significant association (senior vs. mixed: 5.2% vs. 8.4%, *p* = 0.054).

### 3.10. GRADE Evidence Quality Assessment

The overall quality of evidence was evaluated using the GRADE framework, with most outcomes rated low to very low (Supplementary Table S3). Overall major complications received a very low rating (⊕⊝⊝⊝) due to risk of bias, inconsistency, and publication bias. Device failure requiring surgery received a low rating (⊕⊕⊝⊝) due to risk of bias and inconsistency, but no publication bias.

Vestibular complications received a very low rating due to risk of bias, inconsistency, indirectness, and publication bias. However, the adult versus pediatric comparison received a low rating due to the presence of a strong association (RR > 2.00), supporting the upgrade despite limitations. Similarly, the temporal trends comparison received a low rating due to the strong association demonstrating improvement over time. The limitations were mainly attributed to the observational nature of the included studies, heterogeneity, varying definitions, and publication bias; however, consistency across sensitivity analyses provided reassurance regarding the main conclusions.

## 4. Discussion

CI has become a routine procedure for auditory rehabilitation for profoundly deaf patients. However, as with any other procedure, CI has reported various postoperative complications. Awareness of complications and their early anticipation can preserve an expensive device and more importantly, minimize patient morbidity. Numerous studies in the literature have reported on cochlear implant complications; however, the data often are derived from single center experience or specific populations [2,4]. In our study, we aimed to comprehensively assess the rate of complications related to CI and its associated risk factors.

We included a total of 81 studies, the majority of which were cohort in design and encompassed a total of 42,167 patients. The overall incidence of any complication was found to be 13.7% (major complications 5.2%, minor complications 7.8%).

Adults demonstrated higher overall complication rates of 6.8%, with device failures showing a substantial age-related disparity (3.4% in adults versus 1.5% in pediatrics). MPTA surgical approach was associated with lower complication rates of 5.4%, compared to 7.8% of SMA procedures, especially for wound complications. Bilateral implantation was associated with higher complication rates of 6.7% compared to unilateral procedures of 5.1%, particularly for vestibular issues. A significant temporal trend toward outcome improvement over time was observed, with complication rates dropping from 8.9% in the early era (1991–2010) to 4.1% in the recent era (2011–2025), with infection rates showing a particularly marked improvement (from 4.2% to 1.35%).

Our study identified several significant risk factors including the following: older age at implantation (with each additional year increasing complication odds by 2.3%), being an adult, and male gender. Patients with inner ear malformations had twice the risk of complications. Surgical factors, including SMA surgery, bilateral implantation, and longer surgery duration were associated with higher complication rates. Notably, a more recent publication year was associated with 4% reduction in complications, indicating progress in surgical safety and management over time.

Our findings align with previous systematic reviews that have generally reported cochlear implantation as a safe procedure with a low overall complication rate. It is important to note that overall complication rates can vary depending on the definition of complications (major versus minor), the duration of follow-up, and the specific patient populations studied [2,13]. For instance, a systematic review by AlRajhi et al. on CI complications in Saudi Arabia reported overall complication rates ranging from 4–13%, which is consistent with our broader estimate of 13.7%. Notably, both studies identified vestibular symptoms as common postoperative complications [2]. Another systematic review focusing on CI among autoimmune inner ear disease reported a comparable overall complication rate of 12.9% [13].

Device failure requiring surgery, which we identified as the most common major complication at 2.8%, is a well-documented issue. Our rate is in line with the existing literature. A large 30-year review noted a higher overall rate of 4.8%, while another large cohort study reported a rate of 2.9% [8,26]. A recent study analyzing 1431 CIs found that 1.9% required revision for device failure [27]. These figures can vary depending on the device manufacturer and implant generation, with earlier models showing higher failure rates [28,29].

Our general findings identified that adult recipients experienced higher complications compared to pediatric patients, which is consistent with some previous studies particularly for complications such as cochleovestibular issues (tinnitus and vertigo) and overall wound complications [30,31]. However, other research reported higher rates of certain complications, such as device-related problems, in younger patients [32,33,34,35,36].

Regarding risk factors, Laureano et al. reported surgical factors influencing wound complications like operative time and the use of specific approaches [37]. We align with their general findings that surgical approaches play a role in complication rates. Also, similar to our findings, they specifically reported that longer operative times were associated with an increased risk of wound complications. Several studies in the literature addressed the association of inner ear malformations with higher risk of complications [38,39]. This is very similar to our findings that patients with inner ear malformations have twice the risk of complications.

One of the strong findings of our study is the significant temporal improvements in the safety of cochlear implantation. This conclusion is strongly supported by and builds upon the existing literature; for instance, a study found that reimplantation rates decreased from 6.8% before 2000 to 3.2% after 2000, linking this to the increase in surgeon experience and incremental refinements in protocols [40]. The reduction in infection rates, which fell from 4.2% to 1.35% in our analysis, is a key component in the temporal trend we observed in our study.

## 5. Limitations and Implications

Several limitations of this review deserve careful consideration. The evidence base is predominantly observational, consisting largely of retrospective cohort studies, with only a few case-control and cross-sectional designs. This inherently raises concerns about bias, such as selection bias, measured confounding, and information bias due to variability in outcome, ascertainment, and reporting. This methodological limitation is consistent with previous systematic reviews in cochlear imbalance research, where Tang et al. (2024) found that only 40% of included reviews met most of the quality criteria using the Joanna Briggs Institute checklist [41]. This was reflected in the GRADE assessment, where most outcomes were rated very low to low due to serious risk of bias and inconsistency.

Perhaps the most consequential limitation, and one that cuts across nearly every finding in this review, is the lack of standardized complication definitions. There is no universal agreement on what separates a “major” from a “minor” complication, no consistent threshold for diagnosing wound infection, and no shared criterion for when a device failure requires surgical revision. When definitions shift from study to study, pooled estimates inevitably reflect a mixture of clinical and reporting practices rather than a single coherent construct, and no statistical method fully corrects for that. This problem is especially acute for vestibular outcomes. Some studies captured vertigo or dizziness through structured patient questionnaires, others through objective tests such as caloric irrigation or the video head impulse test, and many through informal clinical notation with no defined criteria at all. The pooled vestibular complication rate of 12.4% should therefore be read as a rough approximation spanning a wide spectrum of assessment rigor, not as a precise epidemiological estimate. Addressing this through consensus-based reporting guidelines is arguably the single most impactful step the field could take to improve future evidence synthesis.

Heterogeneity was substantial throughout. The I^2^ of 93.6% for overall complications considerably exceeds the moderate heterogeneity (I^2^ = 55%) reported in prior cochlear implant meta-analyses focused on specific outcomes like postoperative meningitis [42] and signals that the included studies are measuring a genuinely diverse clinical reality rather than a minor sampling variation around a common true effect. Meta-regression reduced residual heterogeneity to I^2^ = 28.6% by accounting for publication year, sample size, mean age, and follow-up duration together explaining 72.4% of between-study variance; however, the remaining unexplained variance reflects subtler differences in surgical technique, patient selection thresholds, device generations, and postoperative protocols that were not consistently reported. Cooper et al. (2025) identified the same structural problem: without agreed-upon denominators, time windows, and outcome labels, cross-study comparisons remaining inherently imprecise [43]. It is also worth noting that the meta-regression associations are ecological in nature; they describe relationships at the study level, not the patient level and should not be interpreted as directly predictive of individual patient risk.

Publication bias was detected for several outcomes, including overall major complications, vertigo, tinnitus, and the early versus recent era comparison. The consistent pattern in trim-and-fill analyses where adjusted estimates were lower than unadjusted ones in every case (e.g., major complications: 4.8% vs. 5.2%; tinnitus: 8.1% vs. 8.9%) suggests that the likely direction of bias is toward overestimation, possibly because studies from high volume centers reporting notable complication events are more likely to be published than routine single center series with unremarkable outcomes. Trim-and-fill correction attenuates but does not eliminate this distortion, and absolute rates throughout this review should be interpreted with that in mind; similar concern were also highlighted in previous meta-analyses [44].

Generalizability also warrants comment. Studies from the United States accounted for nearly a third of the sample, with the remainder drawn from Europe and Asia. Regional differences in patient populations, surgical practices, and healthcare systems could influence outcomes, and it is unlikely that a single pooled estimate captures this diversity without the remainder. Moreover, data granularity also limited deeper analyses. For example, surgeon experience was broadly categorized, and device type was analyzed by the manufacturer without model-level detail, potentially obscuring device-specific risk profiles, an important limitation given the reports of implant survival variability across manufacturers [40,45]. Inconsistent reporting of comorbidities and anatomical variations further restricted exploration of other possible influencing factors.

In addition, the available data permitted only an overall comparison of unilateral and bilateral procedures; the limited number of studies reporting laterality-specific outcomes precluded a more detailed analysis of how implantation laterality influences specific complication types; this should be addressed in future studies.

Collectively, these limitations emphasize the need for standardized definitions, improved methodological rigor, and comprehensive data reporting in future studies. Large, multicenter, prospective cohorts with uniform definitions of complications and detailed documentation of patient, surgical, and device variables are essential to minimize heterogeneity and bias [40,41,43]. Establishing consensus-based reporting guidelines for cochlear implant complications will improve comparability, reduce selective reporting, and strengthen evidence synthesis. Future research should prioritize detailed analyses of common complications such as vertigo, dizziness, and tinnitus to clarify pathophysiology and guide preventive strategies [46,47]. Additionally, addressing publication bias through mandatory trial registration and transparent reporting will enhance data integrity and inform balanced health policy decisions. By acknowledging these limitations and addressing them through standardized, high-quality research, the field can move toward safer, more evidence-based practices in cochlear implantation.

## 6. Conclusions

Based on the available evidence, cochlear implantation appears to be a generally safe procedure; however, given the substantial heterogeneity (I^2^ up to 93.6%) and the predominantly retrospective, observational nature of the included evidence, these conclusions should be interpreted with caution. Identifying and mitigating risk factors, including patient demographics, comorbidities, and surgical approaches, remains clinically relevant, though the strength of evidence supporting individual risk factor associations is limited. Further research with standardized complication reporting, prospective designs, and patient-level data is warranted to strengthen the evidence base.

## Figures and Tables

**Table 1:** Combined and summarized included study characteristics and patient demographics.

Characteristic	Value
Study Characteristics:	
Total studies included	100
Publication period	1991–2025
Study design:	
Cohort studies	92 (92.0%)
Case-control studies	6 (6.0%)
Cross-sectional studies	2 (2.0%)
Geographic distribution:	
United States	32 (32.0%)
China	8 (8.0%)
Germany	6 (6.0%)
Turkey	5 (5.0%)
France	5 (5.0%)
Other countries	44 (44.0%)
Patient Demographics:	
Total patients analyzed	42,167
Sample size per study, median (range)	260 (20–8329)
Age distribution:	
Adults only (≥18 years)	40 (40.0%)
Pediatric only (<18 years)	37 (37.0%)
Mixed population	23 (23.0%)
Gender distribution (*n* = 63 studies) ^a^:	
Male, mean % (range)	52.4% (12.5–59.6%)
Female, mean % (range)	47.6% (40.4–87.5%)
Clinical Characteristics:	
Hearing loss onset (*n* = 82 studies) ^a^:	
Prelingual	48 (58.5%)
Postlingual	29 (35.4%)
Mixed	5 (6.1%)
Hearing loss degree:	
Profound	89 (89.0%)
Severe to profound	11 (11.0%)
Surgical and Device Characteristics:	
Implant manufacturer:	
Cochlear/Nucleus	47 (47.0%)
Med-El	18 (18.0%)
Advanced Bionics	8 (8.0%)
Mixed devices	27 (27.0%)
Surgical approach (*n* = 89 studies) ^a^:	
MPTA	84 (94.4%)
SMA	5 (5.6%)
Bilateral implantation:	
Unilateral procedures	63 (63.0%)
Bilateral included	37 (37.0%)
Follow-up Characteristics:	
Follow-up duration:	
Median, months (range)	36 (0.5–202)
<12 months	15 (15.0%)
12–60 months	65 (65.0%)
>60 months	20 (20.0%)

**Note:** ^a^ Number of studies reporting this characteristic. **Abbreviations:** MPTA, mastoidectomy with posterior tympanotomy approach; SMA, suprameatal approach.

**Table 2:** Pooled complication rates by category across included studies.

Complication Type	Studies Reporting (*n*)	Events/Total Patients	Pooled Estimate % (95% CI)	I^2^ (%)	*p*-Value
Major Complications:					
Overall major complications	*78*	1847/42,167	5.2 (4.1–6.6)	89.2	<0.001
Device failure requiring surgery	*64*	867/38,423	2.8 (2.1–3.7)	78.5	<0.001
Facial nerve paralysis (permanent)	*52*	234/35,891	0.8 (0.5–1.2)	52.3	0.002
Meningitis	*28*	127/28,934	0.4 (0.2–0.7)	45.1	0.008
Electrode displacement/migration	*35*	198/24,567	1.1 (0.7–1.7)	68.9	<0.001
CSF leak/gusher	*45*	892/31,245	2.1 (1.4–3.1)	85.6	<0.001
Minor Complications:					
Overall minor complications	*62*	2134/39,876	7.8 (6.2–9.8)	92.1	<0.001
Wound infection (superficial)	*71*	743/40,523	2.3 (1.8–2.9)	72.4	<0.001
Hematoma/seroma	*43*	312/29,187	1.4 (0.9–2.1)	61.2	<0.001
Transient facial weakness	*38*	245/26,934	1.2 (0.8–1.8)	55.7	0.001
Wound dehiscence	*29*	89/22,156	0.5 (0.3–0.9)	38.2	0.024
Magnet displacement	*31*	178/23,467	0.9 (0.5–1.5)	67.8	<0.001
Vestibular Complications:					
Vertigo/dizziness (any)	*68*	3247/38,912	12.4 (9.8–15.6)	95.3	<0.001
Persistent vertigo (>6 months)	*42*	892/28,734	4.2 (3.1–5.7)	81.2	<0.001
Balance dysfunction	*35*	567/25,891	3.1 (2.2–4.4)	76.8	<0.001
Other Complications:					
Tinnitus (new or worsened)	*28*	1245/21,567	8.9 (6.1–12.8)	88.7	<0.001
Taste disturbance	*18*	287/15,234	2.4 (1.5–3.8)	64.3	<0.001
Chorda tympani injury	*25*	434/19,876	2.8 (1.9–4.1)	73.5	<0.001
Surgical Outcomes:					
Any reoperation	*89*	1923/41,234	6.1 (5.1–7.3)	84.7	<0.001
Revision surgery	*67*	1456/37,891	4.8 (3.9–5.9)	79.2	<0.001
Explantation	*34*	298/26,123	1.4 (0.9–2.1)	58.9	<0.001
Temporal Analysis:					
Early complications (<30 days)	*56*	1567/34,892	5.8 (4.6–7.3)	86.1	<0.001
Late complications (≥30 days)	*48*	2234/32,156	8.9 (7.2–11.0)	89.4	<0.001
Overall Safety Profile:					
Any complication	*94*	4892/41,923	13.7 (11.8–15.9)	93.6	<0.001
Serious adverse events	*72*	2156/39,234	6.8 (5.7–8.1)	87.3	<0.001

**Abbreviations:** CI, confidence interval; CSF, cerebrospinal fluid; I^2^, inconsistency index.

**Table 4:** Risk factors analysis results.

Risk Factor	Category	Studies (*n*)	Patients (*n*)	Univariate Analysis OR (95% CI)	Multivariate Analysis *p*-Value
Patient Characteristics:					
Age at implantation (per year)	Continuous	*45*	*28,456*	1.023 (1.007–1.040)	0.004
Adult vs. pediatric	Categorical	*77*	*34,245*	1.62 (1.23–2.14)	0.001
Male gender	Categorical	*63*	*32,167*	1.21 (1.01–1.44)	0.035
Hearing Loss Characteristics:					
Prelingual vs. postlingual	Categorical	*82*	*35,892*	0.78 (0.64–0.96)	0.019
HL duration (per month)	Continuous	*34*	*22,567*	1.002 (1.000–1.004)	0.048
Inner ear malformations	Categorical	*42*	*25,734*	2.00 (1.44–2.78)	<0.001
Comorbidities:					
Chronic otitis media	Categorical	*35*	*19,823*	1.67 (1.24–2.25)	0.001
Previous otologic surgery	Categorical	*28*	*18,923*	1.50 (1.11–2.03)	0.009
Diabetes mellitus	Categorical	*18*	*12,456*	1.38 (0.96–1.98)	0.082
Surgical Factors:					
MPTA vs. SMA approach	Categorical	*89*	*41,390*	0.69 (0.48–0.99)	0.045
Bilateral implantation	Categorical	*37*	*15,723*	1.32 (1.01–1.71)	0.041
Surgery duration (per hour)	Continuous	*23*	*14,567*	1.15 (1.02–1.30)	0.025
Surgeon experience (senior vs. mixed)	Categorical	*87*	*40,234*	0.62 (0.38–1.01)	0.054
Device Characteristics:					
Med-El vs. Cochlear/Nucleus	Categorical	*65*	*33,501*	1.29 (0.85–1.96)	0.237
Advanced Bionics vs. Cochlear/Nucleus	Categorical	*55*	*28,134*	1.58 (0.91–2.74)	0.104
Perimodiolar vs. straight electrode	Categorical	*34*	*21,789*	0.83 (0.61–1.13)	0.235
Study-Level Factors:					
Publication year (per year)	Continuous	*100*	*42,167*	0.96 (0.94–0.98)	<0.001
Sample size (per 100 patients)	Continuous	*100*	*42,167*	0.98 (0.96–0.99)	0.012
Geographic region:					
Europe vs. North America	Categorical	*63*	*30,801*	1.23 (0.95–1.59)	0.118
Asia vs. North America	Categorical	*60*	*27,125*	1.29 (0.98–1.70)	0.067
Follow-up duration (per month)	Continuous	*82*	*38,234*	1.003 (1.000–1.006)	0.043
Interaction Effects:					
Age × Inner ear malformation	Interaction	*35*	*22,456*	1.45 (1.08–1.95)	0.014
Bilateral × Adult age	Interaction	*25*	*12,789*	1.67 (1.12–2.49)	0.012
Publication year × Region	Interaction	*100*	*42,167*	0.94 (0.89–0.99)	0.031

**Abbreviations:** CI, confidence interval; HL, hearing loss; MPTA, mastoidectomy with posterior tympanotomy approach; OR, odds ratio; SMA, suprameatal approach.

**Table 5:** Temporal patterns and healthcare utilization stratified by age group and implantation era.

Outcome Measure	Studies (*n*)	Patients (*n*)	Mean/Rate (Range)	95% CI	I^2^ (%)	Subgroup Analysis
Device Activation and Setup:						
Time to activation (days)	*67*	*34,567*	32.4 (14–56)	29.8–35.1	76.8	A: 30.2; P: 35.1
Initial programming sessions	*45*	*28,234*	4.2 (2–8)	3.8–4.6	68.2	A: 3.9; P: 4.6
Time to optimal programming (weeks)	*38*	*23,891*	8.7 (4–16)	7.9–9.5	72.4	A: 7.8; P: 9.8
Surgical Recovery:						
Hospital length of stay (days)	*52*	*31,456*	1.8 (1–5)	1.6–2.0	54.3	A: 2.1; P: 1.4
Return to normal activities (days)	*34*	*21,234*	12.6 (7–21)	11.2–14.0	61.7	A: 14.2; P: 10.8
Wound healing time (days)	*29*	*18,567*	10.4 (7–18)	9.6–11.2	43.2	No difference
Temporal Complication Patterns:						
Early complications (<30 days) %	*78*	*39,234*	5.8 (2.1–12.4)	4.9–6.8	82.1	A: 6.8; P: 4.6
Late complications (30 d–1 y) %	*72*	*37,891*	6.2 (2.8–14.2)	5.2–7.4	79.3	A: 7.1; P: 5.1
Very late complications (>1 y) %	*58*	*32,567*	2.7 (0.8–7.3)	2.1–3.5	71.8	A: 3.2; P: 2.1
Specific Temporal Complications:						
Infection onset (median days)	*63*	*35,234*	8.5 (2–28)	7.2–9.8	65.4	Early: 83%
Device failure onset (median months)	*47*	*28,891*	18.7 (1–84)	15.2–22.1	88.9	Trauma: 6.2 mo
Vertigo onset (median days)	*55*	*31,567*	3.2 (0–14)	2.8–3.6	91.2	Immediate: 67%
Functional Outcomes:						
Time to first word recognition (months)	*41*	*25,789*	3.8 (1–12)	3.2–4.4	79.6	A: 2.1; P: 5.8
Time to sentence comprehension (months)	*35*	*22,456*	8.4 (3–24)	7.1–9.7	84.2	A: 5.2; P: 12.1
Hearing improvement (% achieving benefit)	*89*	*40,234*	87.3 (72–96)	85.1–89.5	67.8	A: 89.2; P: 85.1
Quality of Life Measures:						
QOL score improvement (% change)	*28*	*16,789*	68.4 (42–89)	62.1–74.7	76.3	A: 71.2; P: 65.1
Patient satisfaction (% satisfied)	*31*	*18,934*	91.2 (78–98)	88.7–93.7	58.9	A: 92.1; P: 90.2
Return to work/school (% within 1 year)	*24*	*14,567*	76.8 (58–92)	72.3–81.3	69.4	A: 78.9; P: 74.2
Healthcare Resource Utilization:						
Audiologist visits (first year)	*43*	*26,891*	6.8 (4–12)	6.2–7.4	63.7	P: 8.2; A: 5.9
ENT follow-up visits (first year)	*67*	*35,234*	3.4 (2–6)	3.1–3.7	52.1	No difference
Emergency department visits %	*48*	*29,567*	4.2 (1.2–9.8)	3.4–5.1	71.6	A: 5.1; P: 3.1
Revision and Additional Procedures:						
Time to revision surgery (median months)	*56*	*32,123*	24.6 (3–72)	20.8–28.4	82.7	Device: 18.2 mo
Additional procedures per patient	*73*	*37,456*	0.3 (0–1.2)	0.2–0.4	67.3	A: 0.4; P: 0.2
Reimplantation rate %	*89*	*40,567*	4.8 (1.2–12.1)	4.1–5.6	79.8	A: 5.2; P: 4.3
Long-term Success Metrics:						
Device use at 5 years %	*45*	*27,891*	94.2 (87–98)	92.8–95.6	48.7	A: 95.1; P: 93.1
Continued benefit at 5 years %	*41*	*25,234*	91.8 (84–97)	90.1–93.5	52.3	A: 93.2; P: 90.1
No major complications at 5 years %	*38*	*23,567*	88.4 (79–94)	86.2–90.6	61.8	A: 86.9; P: 90.2
Cost and Economic Outcomes:						
Total healthcare costs (first year, USD)	*18*	*12,345*	4820 (3200–7100)	4234–5406	78.9	A: 5200; P: 4380
Cost per QALY gained (USD)	*12*	*8567*	18,450 (12,000–28,000)	15,234–21,666	68.4	A: 16,800; P: 20,300
Temporal Trends by Era:						
Early Era (1991–2010):						
Time to activation (days)	*35*	*12,345*	42.8 (28–84)	38.9–46.7	82.4	-
Hospital stay (days)	*28*	*10,234*	2.8 (1–7)	2.4–3.2	67.8	-
Complication rate %	*35*	*12,345*	8.9 (5.2–15.6)	7.6–10.2	85.3	-
Recent Era (2011–2025):						
Time to activation (days)	*65*	*29,822*	28.2 (14–42)	26.8–29.6	64.2	*p* < 0.001 *
Hospital stay (days)	*47*	*25,678*	1.4 (1–3)	1.2–1.6	45.7	*p* < 0.001 *
Complication rate %	*65*	*29,822*	4.1 (2.1–8.3)	3.6–4.6	71.4	*p* < 0.001 *

**Notes:** * Comparison between early and recent eras using meta-regression, random-effects meta-analysis with subgroup analysis by age group where data available, Healthcare utilization data converted to standardized units where necessary. **Abbreviations:** A, adults; CI, confidence interval; ENT, ear, nose, and throat; I^2^, inconsistency index; P, pediatric; QALY, quality-adjusted life year; QOL, quality of life; USD, United States dollars.

**Table 6:** Egger’s and Begg’s test results with trim-and-fill adjusted estimates and sensitivity analysis outcomes by complication category.

Outcome	Studies (*n*)	Publication Bias Tests	Sensitivity Analyses
		Egger’s Test	Begg’s Test
		t-Stat (*p*-Value)	z-Stat (*p*-Value)
Overall Major Complications	*78*	2.84 (0.006) *	1.92 (0.055)
Device Failure Requiring Surgery	*64*	1.23 (0.223)	0.87 (0.384)
Wound Infections	*71*	0.94 (0.349)	0.72 (0.471)
Facial Nerve Paralysis	*52*	0.67 (0.506)	0.45 (0.653)
Vertigo/Dizziness	*68*	3.67 (<0.001) ***	2.84 (0.005) **
Tinnitus (New/Worsened)	*28*	2.34 (0.027) *	1.67 (0.095)
Any Reoperation	*89*	1.67 (0.098)	1.34 (0.181)
Overall Complications	*94*	2.45 (0.016) *	1.89 (0.059)
Subgroup: Adults vs. Pediatric	*77*	1.89 (0.062)	1.45 (0.147)
Subgroup: Early vs. Recent Era	*100*	3.12 (0.002) **	2.34 (0.019) *

**Note:** * *p* < 0.05, ** *p* < 0.01, *** *p* < 0.001.

## Data Availability

The original contributions presented in this study are included in the article and Supplementary Material. Further inquiries can be directed to the corresponding author.

## Supplementary Materials

The following supporting information can be downloaded at https://www.mdpi.com/article/10.3390/audiolres16040110/s1, Figure S1: Bar plot of the pooled cochlear-implant complication rates; Table S1: Keywords and Search Strategy; Table S2: Risk of Bias Assessment of Included Studies; Table S3: GRADE Evidence Quality Assessment; Table S4: Detailed Complication Breakdown and Sensitivity Analyses.
