# Supplementary material for: Incidence and Risk Factors of Cochlear Implant Complications: A Systematic Review and Meta-Analysis
Source: Audiol Res. 2026 Jul 29;16(4):110. doi: 10.3390/audiolres16040110 (PMC13509191; doi:10.3390/audiolres16040110)
Supplement: Supplementary file 1 [file audiolres-16-00110-s001.zip › Figure S1.pdf]

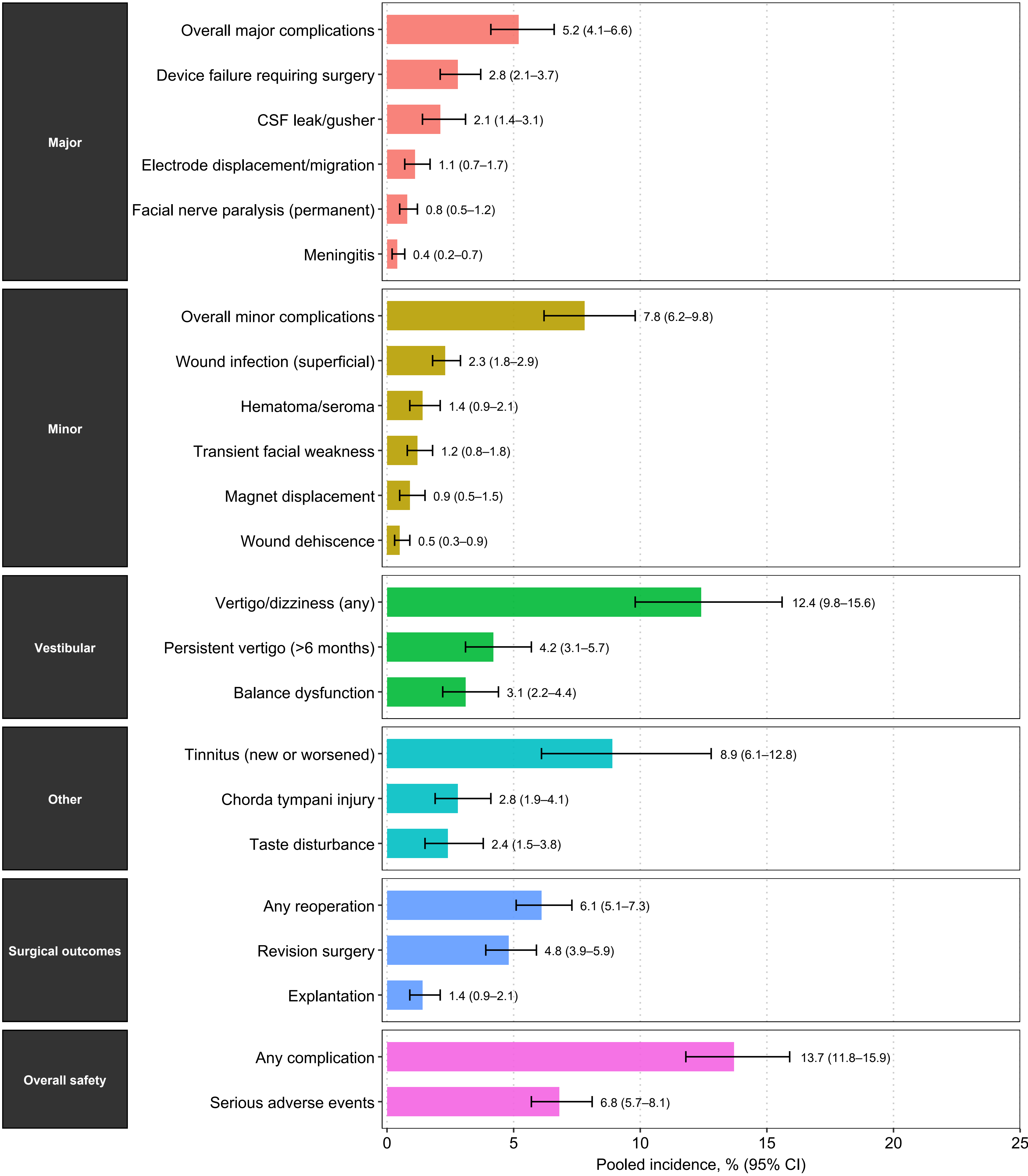

Major
Overall major complications
5.2 (4.1–6.6)
Device failure requiring surgery
2.8 (2.1–3.7)
CSF leak/gusher
2.1 (1.4–3.1)
Electrode displacement/migration
1.1 (0.7–1.7)
Facial nerve paralysis (permanent)
0.8 (0.5–1.2)
Meningitis
0.4 (0.2–0.7)
Minor
Overall minor complications
7.8 (6.2–9.8)
Wound infection (superficial)
2.3 (1.8–2.9)
Hematoma/seroma
1.4 (0.9–2.1)
Transient facial weakness
1.2 (0.8–1.8)
Magnet displacement
0.9 (0.5–1.5)
Wound dehiscence
0.5 (0.3–0.9)
Vestibular
Vertigo/dizziness (any)
12.4 (9.8–15.6)
Persistent vertigo (>6 months)
4.2 (3.1–5.7)
Balance dysfunction
3.1 (2.2–4.4)
Other
Tinnitus (new or worsened)
8.9 (6.1–12.8)
Chorda tympani injury
2.8 (1.9–4.1)
Taste disturbance
2.4 (1.5–3.8)
Surgical outcomes
Any reoperation
6.1 (5.1–7.3)
Revision surgery
4.8 (3.9–5.9)
Explantation
1.4 (0.9–2.1)
Overall safety
Any complication
13.7 (11.8–15.9)
Serious adverse events
6.8 (5.7–8.1)
0
5
10
15
20
25
Pooled incidence, % (95% CI)
