# Supplementary material for: Incidence and Risk Factors of Cochlear Implant Complications: A Systematic Review and Meta-Analysis
Source: Audiol Res. 2026 Jul 29;16(4):110. doi: 10.3390/audiolres16040110 (PMC13509191; doi:10.3390/audiolres16040110)
Supplement: Supplementary file 1 [file audiolres-16-00110-s001.zip › Supplementary Table S1 .pdf]

Supplementary Table S1: Keywords and Search Strategy

Database	Used Strategy	Numbers of results
Pubmed	("Cochlear Implant*" OR "Cochlear Prosthes*" OR "Auditory Prosthes*" OR "Cochlear Device*" OR "Auditory Implant*" OR "Hearing Implant*") AND ("Postoperative Complications"[MeSH Terms] OR "Surgical Wound Infection"[MeSH Terms] OR "Device Failure"[MeSH Terms] OR "Treatment Failure"[MeSH Terms] OR "Adverse Effects"[Subheading] OR "Complication*" OR "Adverse Event*" OR "Surgical Complication*" OR "Infection*" OR "Failure*") AND ("Incidence" OR "Risk Factors"[MeSH Terms] OR "Predictors" OR "Prevalence"[MeSH Terms] OR "Epidemiology"[Subheading] OR "Frequency" OR "Occurrence")	1262 results
Web of Science	TS=(Cochlear Implant* OR Cochlear Prosthes* OR Auditory Prosthes* OR Cochlear Device* OR Auditory Implant* OR Hearing Implant*) AND TS=(Postoperative Complication* OR Surgical Wound Infection OR Device Failure* OR Treatment Failure* OR Adverse Effect* OR Complication* OR Adverse Event* OR Surgical Complication* OR Infection* OR Failure*) AND TS=(Incidence OR Risk Factor* OR Predictor* OR Prevalence OR Epidemiology OR Frequency OR Occurrence)	929 results
Embase	('cochlear implant*' OR 'cochlear prosthes*' OR 'auditory prosthes*' OR 'cochlear device*' OR 'auditory implant*' OR 'cochlea prosthesis'/exp OR 'cochlea prosthesis') AND (postoperative AND complication*) OR ('surgical wound infection'/exp OR 'surgical wound infection') OR (surgical AND ('wound'/exp OR wound) AND ('infection'/exp OR infection)) OR ('device'/exp OR device) AND (failure* OR 'treatment'/exp OR treatment) OR (failure* OR adverse) OR (effect* OR complication* OR adverse) OR (event* OR surgical) OR (complication* OR infection* OR failure*) AND ('incidence'/exp OR incidence OR 'risk'/exp OR risk) AND (factor* OR predictor* OR 'prevalence'/exp OR prevalence OR 'epidemiology'/exp OR epidemiology OR 'frequency'/exp OR frequency OR occurrence)	1071
Scoups	Article title-Abstract-keywords ("Cochlear Implant*" OR "Cochlear Prosthes*" OR "Auditory Prosthes*" OR "Cochlear Device*" OR "Auditory Implant*" OR "Hearing Implant*") AND Article title-Abstract-keywords ("Postoperative Complication*" OR "Surgical Wound Infection*" OR "Device Failure*" OR "Treatment Failure*" OR "Adverse Effect*" OR "Complication*" OR "Adverse Event*" OR "Surgical Complication*" OR "Infection*" OR "Failure*) AND Article title-Abstract-keywords ("Incidence" OR "Risk Factor*" OR "Predictor*" OR "Prevalence" OR "Epidemiology" OR "Frequency" OR "Occurrence")	1195
