# Supplementary material for: Incidence and Risk Factors of Cochlear Implant Complications: A Systematic Review and Meta-Analysis
Source: Audiol Res. 2026 Jul 29;16(4):110. doi: 10.3390/audiolres16040110 (PMC13509191; doi:10.3390/audiolres16040110)
Supplement: Supplementary file 1 [file audiolres-16-00110-s001.zip › Supplementary Table S2 (1).pdf]

Supplementary Table S2: Risk of Bias Assessment of Included Studies.

Study Name	Selection (Max 4 Stars)	Comparability (Max 2 Stars)	Outcome (Max 3 Stars)	Total Score (Max 9 Stars)
Alqahtani et al. 2025	★★★☆	★☆	★★★	7
Ketterer et al. 2024	★★★☆	★★	★★★	8
Al Mansoury et al. 2024	★★☆☆	★☆	★☆☆	4
Molaei Sisakht et al. 2023	★★★☆	★☆	★★☆	6
Rotlevi et al. 2023	★★★☆	★☆	★☆☆	5
Mathews et al. 2023	★★★☆	★☆	★★★	7
Shu et al. 2022	★★★☆	★☆	★★★	7
Devroede et al. 2020	★★★☆	★★	★★★	8
Kelsall et al. 2019	★★★☆	★★	★★★	8
Sladen et al. 2017	★★★☆	★★	★★★	8
Dillon et al. 2017	★★★☆	★★	★★★	8
Zuniga et al. 2016	★★★☆	★★	★★☆	7
Todt et al. 2016	★★★☆	★★	★★★	8
Gawęcki et al. 2016	★★★☆	★★	★★★	8
Alcas & Salazar 2016	★★★☆	★☆	★★☆	6
Kim et al. 2016	★★★☆	★★	★★★	8
Arts et al. 2015	★★★☆	★★	★★★	8
Darlong et al. 2015	★★★☆	★☆	★★☆	6
Mosnier et al. 2015	★★★☆	★★	★★★	8
Terry et al. 2015	★★★☆	★★	★★★	8
Francis et al. 2015	★★★☆	★★	★★★	8
Farinetti et al. 2014	★★★☆	★☆	★★☆	6
Aynehchi et al. 2014	★★★☆	★★	★★★	8

Company et al. 2014	★★★☆	★★	★★★	8
Vincenti et al. 2014	★★★☆	★★	★★★	8
Afsharpaiman et al. 2014	★★★☆	★☆	★★☆	6
Li et al. 2014	★★★☆	★★	★★★	8
Mistry et al. 2014	★★★☆	★★	★★☆	7
Low et al. 2013	★★★☆	★★	★★★	8
Tarkan et al. 2013	★★★☆	★★	★★★	8
Kunda et al. 2013	★★★☆	★★	★★☆	7
Jeppesen & Faber 2013	★★★☆	★★	★★★	8
Chen et al. 2013	★★★☆	★★	★★★	8
Chang et al. 2013	★★★☆	★★	★★★	8
Hansen et al. 2013	★★★☆	★★	★★★	8
Ciorba et al. 2012	★★★☆	★★	★★★	8
Brito et al. 2012	★★★☆	★★	★★★	8
Coordes et al. 2012	★★★☆	★★	★★★	8
Hashemi et al. 2012	★★★☆	★☆	★★☆	6
Shinghal et al. 2012	★★★☆	★★	★★★	8
Zernotti et al. 2012	★★★☆	★★	★★☆	7
Angeli et al. 2012	★★★☆	★★	★★★	8
Raghunandhan et al. 2011	★★★☆	★☆	★★☆	6
Lescanne et al. 2011	★★★☆	★★	★★★	8
Carlson et al. 2011	★★★☆	★★	★★★	8
Song et al. 2011	★★★☆	★★	★★☆	7
Lalwani & Cohen 2011	★★★☆	★★	★★★	8
Qiu et al. 2011	★★★☆	★★	★★★	8

Stamatiou et al. 2011	★★★☆	★★	★★★	8
Coelho et al. 2011	★★★☆	★★	★★★	8
Hansen et al. 2010	★★★☆	★★	★★★	8
Wang et al. 2010	★★★☆	★☆	★★☆	6
Hou et al. 2010	★★★☆	★★	★★★	8
Loundon et al. 2010	★★★☆	★★	★★★	8
Wagner et al. 2010	★★★☆	★★	★★★	8
Zeitler et al. 2009	★★★☆	★★	★★★	8
Ding et al. 2009	★★★☆	★☆	★★☆	6
Krause et al. 2009	★★★☆	★★	★★★	8
Cullen et al. 2008	★★★☆	★★	★★★	8
Rivas et al. 2008	★★★☆	★★	★★★	8
Kim et al. 2008	★★★☆	★★	★★★	8
Venail et al. 2008	★★★☆	★☆	★★★	7
Viccaro et al. 2007	★★★☆	★★	★★★	8
Dodson et al. 2007	★★★☆	★★	★★★	8
Postelmans et al. 2007	★★★☆	★★	★★★	8
Migirov et al. 2006	★★★☆	★★	★★★	8
Smullen et al. 2005	★★★☆	★★	★★★	8
Kubo et al. 2005	★★★☆	★☆	★★☆	6
Ramos et al. 2005	★★★☆	★☆	★★☆	6
Arnoldner et al. 2005	★★★☆	★★	★★★	8
Ray et al. 2004	★★★☆	★★	★★★	8
Cunningham et al. 2004	★★★☆	★★	★★★	8
Green et al. 2004	★★★☆	★★	★★★	8

Fayad et al. 2003	★★★☆	★☆	★★☆	6
Fina et al. 2003	★★★☆	★★	★★★	8
Mylanus et al. 2003	★★★☆	★★	★★★	8
Hehar et al. 2002	★★★☆	★★	★★★	8
Steenerson et al. 2001	★★★☆	★☆	★★☆	6
Proops et al. 1999	★★★☆	★☆	★★☆	6
Cohen et al. 1993	★★★☆	★☆	★★★	7
Webb et al. 1991	★★★☆	★☆	★★☆	6
