# Supplementary material for: Incidence and Risk Factors of Cochlear Implant Complications: A Systematic Review and Meta-Analysis
Source: Audiol Res. 2026 Jul 29;16(4):110. doi: 10.3390/audiolres16040110 (PMC13509191; doi:10.3390/audiolres16040110)
Supplement: Supplementary file 1 [file audiolres-16-00110-s001.zip › Supplementary Table S3 (2).pdf]

Supplementary Table S3: GRADE Evidence Quality Assessment.

Outcome	Studies (n)	Patients (n)	Pooled Estimate (95% CI)	Risk of Bias	Inconsistency	Indirectness	Imprecision	Publication Bias	Other Considerations	Quality Rating	Confidence in Effect Estimate
**Major Complications:**											
Overall major complications	78	42,167	5.2% (4.1-6.6%)	Serious[1]	Very serious[2]	Not serious	Not serious	Suspected[3]	None	⊕⊖⊖⊖ VERY LOW	Very low confidence that the true effect lies close to the estimate
Device failure requiring surgery	64	38,423	2.8% (2.1-3.7%)	Serious[1]	Serious[4]	Not serious	Not serious	Not detected	None	⊕⊕⊖⊖ LOW	Low confidence that the true effect lies close to the estimate

Facial nerve paralysis (permanent)	52	35,891	0.8% (0.5 - 1.2%)	Serious[1]	Not serious	Not serious	Serious[5]	Not detected	None	⊕⊖⊖⊖ VERY LOW	Very low confidence that the true effect lies close to the estimate
Meningitis	28	28,934	0.4% (0.2 - 0.7%)	Serious[1]	Not serious	Not serious	Serious[5]	Not detected[6]	None	⊕⊖⊖⊖ VERY LOW	Very low confidence that the true effect lies close to the estimate
**Minor Complications:**											
Wound infections	71	40,523	2.3% (1.8 - 2.9%)	Serious[1]	Serious[4]	Not serious	Not serious	Not detected	None	⊕⊕⊖⊖ LOW	Low confidence that the true effect lies close to the

											estimate
Hematoma/seroma	43	29,187	1.4% (0.9-2.1%)	Serious[1]	Serious[4]	Not serious	Serious[5]	Not detected[6]	None	⊕⊖⊖⊖ VERY LOW	Very low confidence that the true effect lies close to the estimate
**Vestibular Complications:**											
Vertigo/dizziness (any)	68	38,912	12.4% (9.8-15.6%)	Serious[1]	Very serious[2]	Serious[7]	Not serious	Suspected[3]	None	⊕⊖⊖⊖ VERY LOW	Very low confidence that the true effect lies close to the estimate
Persistent vertigo (>6 months)	42	28,734	4.2% (3.1-5.7%)	Serious[1]	Very serious[2]	Not serious	Not serious	Not detected[6]	None	⊕⊖⊖⊖ VERY LOW	Very low confidence that the true effect lies

											close to the estimate
**Other Complications:**											
Tinnitus (new or worsened)	28	21,567	8.9% (6.1 - 12.8%)	Serious[1]	Very serious[2]	Serious[7]	Not serious	Not detected[6]	None	⊕⊖⊖⊖ VERY LOW	Very low confidence that the true effect lies close to the estimate
**Surgical Outcomes:**											
Any reoperation	89	41,234	6.1% (5.1 - 7.3%)	Serious[1]	Very serious[2]	Not serious	Not serious	Not detected	None	⊕⊖⊖⊖ VERY LOW	Very low confidence that the true effect lies close to the estimate
Revision surgery	67	37,891	4.8% (3.9 -	Serious[1]	Very serious[2]	Not serious	Not serious	Not detected	None	⊕⊖⊖⊖ VE	Very low confidence that

			5.9%)							RY LOW	the true effect lies close to the estimate
**Overall Safety Profile:**											
Any complication	94	41,923	13.7% (11.8-15.9%)	Serious[1]	Very serious[2]	Not serious	Not serious	Suspected[3]	None	⊕⊖⊖⊖ VERY LOW	Very low confidence that the true effect lies close to the estimate
**Subgroup Comparisons:**											
Adults vs pediatric complications	77	34,245	RR: 1.62 (1.18-2.23)	Serious[1]	Very serious[2]	Not serious	Not serious	Not detected	Strong association[8]	⊕⊕⊖⊖ LOW	Low confidence that adults have higher complication rates

MPTA vs SMA approach	89	41, 390	RR: 0.69 (0.48-0.99)	Serious[1]	Serious[4]	Not serious	Serious[9]	Not detected	None	⊕⊖⊖⊖ VERY LOW	Very low confidence in surgical approach comparison
Early vs recent era	100	42, 167	RR: 2.17 (1.78-2.65)	Serious[1]	Very serious[2]	Not serious	Not serious	Not detected	Strong association[8]	⊕⊕⊖⊖ LOW	Low confidence that outcomes improved over time

GRADE Quality Ratings: *⊕⊕⊕⊕ HIGH: Very confident that the true effect lies close to the estimate of effect, ⊕⊕⊕⊖ MODERATE: Moderately confident in the effect estimate, ⊕⊕⊖⊖ LOW: Confidence in the effect estimate is limited, ⊕⊖⊖⊖ VERY LOW: Very little confidence in the effect estimate.* ***Footnotes:*** *[1] Risk of bias concerns: Most studies were observational cohorts with potential selection bias, lack of blinding, and inconsistent outcome definitions, [2] Very serious inconsistency: I^2 >75% with wide prediction intervals and significant subgroup differences, [3] Suspected publication bias: Asymmetric funnel plot and small study effects detected by Egger's test (p<0.10), [4] Serious inconsistency: I^2 50-75% with moderate heterogeneity across studies, [5] Serious imprecision: Wide confidence intervals due to low event rates; optimal information size not met, [6] Publication bias not detected: Symmetric funnel plot and non-significant Egger's test (p>0.10), [7] Serious indirectness: Outcome definitions varied significantly across studies (e.g., vertigo assessment methods), [8] Strong association: Large effect size (RR >2 or <0.5) supporting upgrade consideration, [9] Serious imprecision: Confidence interval includes null effect and clinically important difference* ***Abbreviations:*** *CI, confidence interval; GRADE, Grading of Recommendations Assessment, Development and Evaluation; I^2, inconsistency index; MPTA, mastoidectomy with posterior tympanotomy approach; RR, relative risk; SMA, suprameatal approach.*
