# Supplementary material for: Incidence and Risk Factors of Cochlear Implant Complications: A Systematic Review and Meta-Analysis
Source: Audiol Res. 2026 Jul 29;16(4):110. doi: 10.3390/audiolres16040110 (PMC13509191; doi:10.3390/audiolres16040110)
Supplement: Supplementary file 1 [file audiolres-16-00110-s001.zip › Supplementary Table S4 (1).pdf]

Supplementary Table S4: Detailed Complication Breakdown and Sensitivity Analyses.

Complication Subtype	Studies (n)	Patients (n)	Events (n)	Pooled Rate (95% CI)	Heterogeneity Analysis I^2 (%)	Influence Analysis (τ^2)	Subgroup Effects (95% PI)
**Surgical Site Complications:**							
Skin flap necrosis	18	14,567	89	0.7% (0.4-1.2)	52.1	0.076	0.1-2.8%
Hematoma	43	29,187	312	1.4% (0.9-2.1)	61.2	0.134	0.2-5.1%
Seroma	23	17,834	156	1.0% (0.6-1.6)	48.9	0.089	0.1-3.2%
Wound dehiscence	29	22,156	89	0.5% (0.3-0.9)	38.2	0.067	0.1-2.1%
**Device-Related Complications:**							
Electrode migration/displacement	35	24,567	198	1.1% (0.7-1.7)	68.9	0.156	0.2-4.2%
Magnet displacement	31	23,467	178	0.9% (0.5-1.5)	67.8	0.143	0.1-3.8%
Receiver/stimulator failure	41	28,934	234	1.0% (0.7-1.5)	54.7	0.098	0.2-3.9%
**Neurological Complications:**							
Chorda tympani injury	25	19,876	434	2.8% (1.9-4.1)	73.5	0.187	0.4-9.1%
Facial nerve stimulation	34	23,567	567	3.2% (2.1-4.8)	79.4	0.234	0.5-11.2%
Transient facial weakness	38	26,934	245	1.2% (0.8-1.8)	55.7	0.112	0.2-4.3%
**Inner Ear Complications:**							

CSF leak/gusher	45	31,245	892	2.1% (1.4-3.1)	85.6	0.234	0.3-8.9%
Pneumolabyrinth	12	8934	67	0.8% (0.4-1.5)	58.3	0.098	0.1-3.4%
Perilymphatic fistula	8	6234	34	0.6% (0.3-1.2)	42.1	0.067	0.1-2.8%
**Vestibular Complications (Detailed):**							
Acute vertigo (<7 days)	52	34,567	1,823	8.9% (6.8-11.6)	92.3	0.456	1.2-29.4%
Chronic vertigo (>6 months)	42	28,734	892	4.2% (3.1-5.7)	81.2	0.298	0.7-15.3%
BPPV (confirmed)	15	12,456	234	2.4% (1.5-3.8)	64.2	0.156	0.4-8.9%
**Infectious Complications (Detailed):**							
Superficial wound infection	65	37,892	612	1.9% (1.4-2.6)	68.7	0.145	0.3-6.2%
Deep wound infection	28	23,456	134	0.7% (0.4-1.2)	51.9	0.089	0.1-3.1%
Meningitis	28	28,934	127	0.4% (0.2-0.7)	45.1	0.067	0.1-2.3%
**Revision Surgery Evaluation:**							
Device-related revision	67	37,891	1,234	3.8% (3.0-4.8)	76.4	0.198	0.6-12.1%
Complication-related revision	45	29,567	567	2.2% (1.6-3.0)	69.3	0.167	0.4-7.8%
Elective upgrade revision	23	18,234	234	1.5% (1.0-2.3)	58.9	0.123	0.2-5.4%

Abbreviations: *A, adult; BPPV, benign paroxysmal positional vertigo; CI, confidence interval; CSF, cerebrospinal fluid;* I^2*, inconsistency index; P, pediatric; PI, prediction interval;* τ^2*, tau-squared (between-study variance).*
